# The association between activity patterns and malignant neoplasms of female genital organs: a prospective cohort study of UK biobank

**DOI:** 10.3389/fendo.2026.1845878

**Published:** 2026-05-25

**Authors:** Xuebing Li, Hang Yu, Yubing Teng, Huanyu Guo, Yuqing Liu, Dandan Zhang, Tianshu Han

**Affiliations:** 1 The First Affiliated Hospital of Harbin Medical University, Harbin, Heilongjiang, China; 2 Harbin Medical University, Harbin, Heilongjiang, China

**Keywords:** circadian rhythm, endocrine regulation, female reproductive cancers, melatonin, metabolic health, physical activity timing, prospective cohort study

## Abstract

**Objective:**

The role of physical activity timing in the development and progression of malignant neoplasms of the female reproductive system remains to be elucidated. Malignant neoplasms of female genital organs include those of the vulva, vagina, cervix, uterine corpus, uterus (part unspecified, site not further defined), ovaries, placenta, and other unspecified sites. This study aimed to investigate the association between patterns of physical activity and the risk of malignant neoplasms of the female reproductive system.

**Methods:**

This study was reported in accordance with the STROBE guidelines for observational cohort studies. This research has been conducted using the UK Biobank Resource under Application ID: 103547. This study included 49,540 female participants with valid accelerometer data from the UK Biobank at baseline. Physical activity patterns were defined based on the timing of moderate-to-vigorous physical activity (MVPA) occurrence throughout the day. According to the timing of MVPA, participants were categorized into four groups: morning, evening, mixed-time, and midday-afternoon groups, with the morning group serving as the reference. Cox proportional hazards models were employed to analyze the association between physical activity patterns and the incidence of malignant neoplasms of female genital organs.

**Results:**

During a median follow-up of 12.6 years, 419 incident cases of malignant neoplasms of female genital organs were recorded. After adjusting for potential confounding factors, compared to women who engaged in morning exercise, those in the evening and mixed-time groups exhibited a significantly higher risk of developing reproductive system malignancies (Evening group: hazard ratio [HR] = 1.530, 95% confidence interval [CI]: 1.006–2.326; Mixed-time group: HR = 1.406, 95% CI: 1.026–1.928; *P* for trend <0.05).

**Conclusions:**

Compared to the morning group, engaging in MVPA during the evening or at mixed times was associated with an elevated risk of malignant neoplasms of the female reproductive system. This association was not observed in the midday-afternoon group. Furthermore, no statistically significant associations were found between activity patterns and specific types of female genital organ malignancies.

## 1 Introduction

The physiological function and pathological status of the female reproductive system are continuously modulated by the synergistic interaction between the endocrine and metabolic systems. Disruption of this intricate balance may precipitate a spectrum of gynecological disorders. The development and progression of malignant neoplasms of female genital organs have been closely linked to hormonal dysregulation and metabolic abnormalities. In female populations, hormonal imbalance and metabolic disturbances not only increase the risk of benign gynecological diseases but are also recognized as key drivers in the pathogenesis of malignancies such as ovarian, endometrial, and cervical cancers (1–4). In interventions targeting hormone- and metabolism-related disorders, physical activity—a key modifiable lifestyle factor—has increasingly shown beneficial effects on female reproductive health. Existing evidence indicates that regular physical activity can improve the reproductive system microenvironment through multiple pathways: For hormone-related diseases, moderate-to-vigorous activity (e.g., 150 minutes of brisk walking per week) can reduce adipose tissue accumulation, decrease peripheral estrogen synthesis, and thereby mitigate the risk of endometrial hyperplasia (5); For individuals with metabolic abnormalities, long-term exercise can enhance insulin sensitivity, lower IGF-1 levels, and inhibit the activation of tumor cell proliferative signaling pathways (6). Cohort studies involving high-risk populations for endometrial cancer have shown that engaging in high-intensity activity ≥5 times per week was associated with a 28% reduction in relative risk compared to never or rarely engaging in such activity (7); Studies on ovarian cancer suggest that long-term adherence to leisure-time physical activity is associated with a 20%-24% risk reduction (8). Additionally, the modulatory effects of activity patterns on immune function can enhance the clearance of HPV infections, indirectly reducing the risk of cervical cancer progression. Nevertheless, existing studies on the association between activity patterns and malignant neoplasms of female genital organs remain limited. For instance, existing studies predominantly focus on the total volume of physical activity, with insufficient attention given to the finer dimensions of activity patterns.

Therefore, utilizing the large-scale cohort data from the UK Biobank, this study aims to systematically evaluate the association between moderate-to-vigorous physical activity (MVPA) at different times of day and the incidence risk of malignant neoplasms of female genital organs (e.g., ovarian, endometrial, cervical cancers). By refining the temporal dimension of activity [morning (05:00-11:00), midday-afternoon (11:00-17:00), and evening (17:00-24:00) groups; participants were placed in a mixed-time group if their daily total MVPA across all three time windows was <50%], and combining with diagnoses of malignant neoplasms of female genital organs recorded using the International Classification of Diseases, Tenth Revision (ICD-10) coding system within the UK Biobank, this study seeks to clarify the quantitative association characteristics between activity timing and different malignancies. Furthermore, extensive adjustment for socioeconomic, lifestyle, and biological confounding factors was performed to reduce bias and enhance the accuracy of the results. This study is expected to be the first to reveal the association mechanism between the often-overlooked dimension of “activity timing” and malignant neoplasms of female genital organs, providing novel scientific evidence for developing individualized cancer-preventive exercise prescriptions in clinical settings and optimizing public health intervention strategies for female reproductive health.

## 2 Materials and methods

### 2.1 Study population

**Figure 1:**
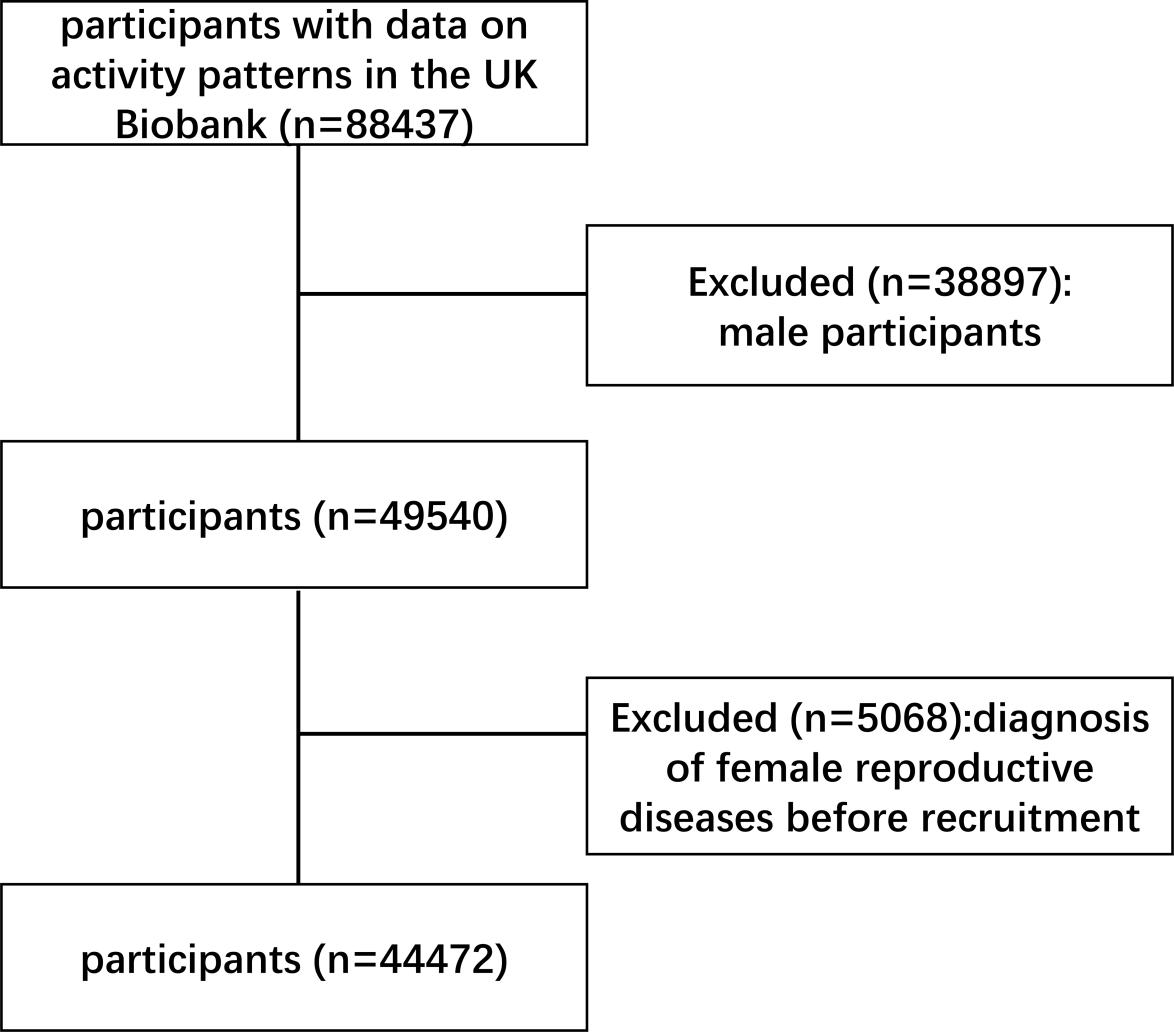
Flow chart of the study population. Malignant tumors of female reproductive organs include malignant tumors of the vulva, vagina, uterine cervix, corpus uteri, unspecified uterine malignancy, malignant tumors of the ovary, other and unspecified malignant tumors of female reproductive organs, and malignant tumors of the placenta. Diagnoses were documented using the International Classification of Diseases, 10th Revision (ICD-10) coding system, including vulvar malignant tumor (C51), vaginal malignant tumor (C52), cervical malignant tumor (C53), corpus uteri malignant tumor (C54), unspecified uterine malignant tumor (C55), ovarian malignant tumor (C56), other and unspecified malignant tumors of female reproductive organs (C57), and placental malignant tumor (C58).

Data for this study were sourced from the UK Biobank study, a large-scale population-based cohort that recruited 502,411 participants aged 37–73 years (response rate: 5.5%). Participants underwent assessments at 22 assessment centers across England, Wales, and Scotland between 2006 and 2010. Between 2013 and 2015, approximately 240,000 invitation letters were randomly sent to a subset of participants to measure physical activity (PA) using accelerometers, with a response rate of 44%. Ultimately, measurement devices were issued to 106,053 participants, and data from 92,091 participants (including 49,540 women) were included in the initial analysis. For this specific study, participants with a pre-existing diagnosis of malignant neoplasms of female genital organs at baseline were excluded, resulting in 44,472 female participants with valid activity pattern data for subsequent in-depth analysis. Furthermore, to explore associations between specific malignant neoplasms of female genital organs (including malignancies of the cervix, uterine corpus, ovaries, and other/unspecified female genital organs) and different dimensions of activity patterns, separate cohorts were constructed; in the analysis of each cohort, participants with a pre-existing diagnosis of the corresponding target disease at baseline were excluded (specific process shown in **Figure 1**). This study received approval from the North West Multi-Centre Research Ethics Committee and the Institutional Review Board of the Tulane University Biomedical Committee in New Orleans, Louisiana, USA. All participants provided written informed consent (9).

### 2.2 Exposure assessment

Physical activity (PA) data for the 49,540 female UK Biobank participants in this study were collected using a 24-hour wrist-worn accelerometer (Axivity AX3). Data indicated no significant differences in baseline demographic or health-related characteristics between participants who accepted and those who refused accelerometer measurement. Participants were instructed to wear the accelerometer on the dominant wrist for 7 consecutive days while adhering to their habitual daily routines. Periods of ≥1 consecutive hour with no activity were defined as non-wear time; data for such periods were imputed based on wear-time data from the same time window on different days. Participants with any 1-hour period lacking PA data within a 24-hour cycle were excluded. Detailed procedures for data processing and analysis have been published elsewhere (9).

Consistent with previous research (10, 11), this study focused on higher-intensity physical activity, particularly moderate-to-vigorous physical activity (MVPA), to clearly and stably identify activity patterns. MVPA is widely validated as an effective metric for assessing physical activity, typically referring to activities requiring moderate to vigorous effort and accompanied by a significant increase in heart rate (12). Light-intensity physical activity, often associated with walking or sedentary behavior, may interfere with the analysis of the more pertinent temporal distribution of beneficial activity and was therefore not included in this study (11).

Furthermore, the UK Biobank accelerometer expert working group processed the raw accelerometer data (Field ID: 90001) to generate physical activity intensity data. This data was provided at 5-second epochs (Field ID: 90004) using milligravity (mg) as the unit for average vector magnitude, with raw accelerometer signals calibrated to gravity standards. Moderate-intensity physical activity was defined as epochs in which >80% of 5-second average acceleration values fell within 100–400 mg over a 5-minute interval (12).; vigorous-intensity activity was defined as epochs with 5-second average acceleration exceeding 400 mg (13).

Aligning with prior studies (10, 11), and considering the diurnal lifestyle patterns of the study population, where frequent nocturnal activity is often associated with sleep disorders, individuals with excessive nocturnal activity were excluded (i.e., participants whose cumulative PA between 01:00–04:00 accounted for ≥10% of total activity). The study utilized 2-hour intervals as the basic time window, except for the 21:00–24:00 period which used a 3-hour interval, to balance sample size and data accuracy.

Previous attempts to categorize activity timing using average acceleration data resulted in only 0.74%, 10.1%, and 0.70% of participants being classified into morning, midday-afternoon, and evening groups, respectively, with the remaining 88.5% classified into a mixed-time group (11). Similar to previous studies (10, 11), participants were categorized into four groups based on the timing of moderate-to-vigorous physical activity (MVPA) using a 50% allocation method. Briefly, the day was divided into three non-overlapping time windows: morning (05:00–11:00), midday-afternoon (11:00–17:00), and evening (17:00–24:00). For each participant, the proportion of total daily MVPA accumulated in each time window was calculated. If ≥50% of total daily MVPA occurred within a single window, the participant was assigned to the corresponding timing group (morning, midday-afternoon, or evening). If no single window contained ≥50% of total daily MVPA (i.e., MVPA was distributed across multiple periods without a dominant time window), the participant was classified into the mixed-timing group. This study used the morning group as the reference group.

As the core of the study was to analyze the specific time of day when exercise occurred, the focus was on exercise patterns. Accordingly, the daily total exercise time was calculated by summing the minutes of moderate and vigorous physical activity between 05:00 and 24:00, divided by the wear time (i.e., valid monitoring time). Notably, the daily total MVPA time was not included as an exposure metric but was treated as an adjustment variable in subsequent analyses.

In summary, besides excluding participants with “any 1-hour period lacking PA data within a 24-hour cycle” and those with “excessive nocturnal activity (cumulative PA ≥10% between 01:00–04:00),” participants with unreliable or invalid accelerometer measurement data were also excluded.

### 2.3 Ascertainment of main outcomes

Malignant neoplasms of female genital organs in our study included malignancies of the vulva, vagina, cervix, uterine corpus, uterus (part unspecified), ovaries, other and unspecified female genital organs, and placenta. Uterine corpus was defined as malignant neoplasms of the corpus uteri (ICD-10 C54). Unspecified uterus referred to malignant neoplasms of the uterus with no specified anatomical site (ICD-10 C55). Both categories were retained to comprehensively ascertain all uterine malignancies. Following prior research (9), outcomes were ascertained through hospital inpatient records containing admission and diagnosis data sourced from the Hospital Episode Statistics for England, the Scottish Morbidity Record, and the Patient Episode Database for Wales. Diagnoses were recorded using the International Classification of Diseases, Tenth Revision (ICD-10) codes: C51 (malignant neoplasm of vulva), C52 (malignant neoplasm of vagina), C53 (malignant neoplasm of cervix uteri), C54 (malignant neoplasm of corpus uteri), C55 (malignant neoplasm of uterus, part unspecified), C56 (malignant neoplasm of ovary), C57 (malignant neoplasm of other and unspecified female genital organs), and C58 (malignant neoplasm of placenta). Follow-up person-years were calculated from the date of the assessment center visit until the event of interest (date of first reported female genital disease) or a censoring event (date of death or September 30, 2021), whichever occurred first.

### 2.4 Covariates

We controlled for a range of potential confounding factors, including age (years), ethnicity (White, Mixed, Asian or Asian British, Black or Black British, Chinese, or Other ethnic group), education (College or University degree, A levels/AS levels or equivalent, O levels/GCSEs or equivalent, CSEs or equivalent, NVQ or HND or HNC or equivalent), Townsend deprivation index, body mass index (BMI), smoking status (never, former, current), alcohol consumption frequency (daily or almost daily, three or four times a week, once or twice a week, one to three times a month, only on special occasions, never), daily total exercise time (minutes), total weekly metabolic equivalent of task (MET) minutes for all activities (minutes), and history of diseases including diabetes, hypertension, and chronic obstructive pulmonary disease (COPD).

### 2.5 Statistical analysis

As this study utilized the UK Biobank, a large population-based prospective cohort, no formal *a priori* sample size calculation was performed at the design stage. Instead, we included all eligible participants with valid accelerometer data to maximize statistical power. Statistical power in Cox proportional hazards models is primarily determined by the number of events (incident cases) rather than the total sample size. In this study, a total of 419 incident cases of female genital malignant neoplasms were observed during follow-up. Based on the number of covariates included in the fully adjusted model, the events per variable (EPV) was above the commonly recommended threshold of 10, indicating that the sample size provided adequate statistical power to detect meaningful associations. The long median follow-up duration of 12.6 years further ensured sufficient event accumulation for reliable effect estimation. Missing data for baseline covariates were handled as follows: for categorical variables, missing values were coded as a separate category; for continuous variables, missing values were imputed using the sample mean. Most covariates had minimal missing data (<1%), except for MET (17.3%). Baseline characteristics regarding demographics, anthropometrics, lifestyle, and health status are presented as mean (standard deviation) and number (percentage). General linear models and chi-square tests were used to compare differences in baseline characteristics across activity pattern groups. Cox proportional hazards models, with follow-up time as the time scale, were employed to calculate hazard ratios (HRs) and 95% confidence intervals (CIs) for the associations between activity patterns and the incidence of malignant neoplasms of female genital organs. Additionally, we used Cox proportional hazards models to assess the relationship between activity patterns and the risk of specific malignant neoplasms of female genital organs; however, certain diseases (malignancies of vulva, vagina, uterus part unspecified, placenta) had nearly zero outcome counts in given activity patterns and were not analyzed further. Furthermore, stratified analyses were conducted by the following factors: ethnicity (White or non-White), education (University degree, below University degree, none of the above), Townsend deprivation index (<median or ≥median), smoking (never, former, current), and alcohol frequency (daily or almost daily, three or four times a week, once or twice a week, one to three times a month, only on special occasions, never) to evaluate potential multiplicative interactions between activity patterns and these factors by adding interaction terms to the Cox models. Four sensitivity analyses were performed to assess the robustness of our findings. The first and second analyses excluded participants diagnosed with malignant neoplasms of the genital organs within one year and two years of follow-up, respectively, to examine whether early diagnosis during follow-up influenced the results. The third analysis excluded participants with diabetes. The fourth analysis excluded participants with COPD. Statistical analyses were performed using R version 4.2.2, with a two-sided P-value <0.05 considered statistically significant.

## 3 Results

### 3.1 Baseline characteristics of participants by activity pattern

**Table 1** presents the baseline differences in demographic, anthropometric, lifestyle, and disease history characteristics among the 44,472 participants according to their activity patterns. Compared to participants who engaged in MVPA in the morning, those with a mixed-time MVPA pattern were more likely to be non-White, had a higher proportion of University graduates or above, a higher Townsend deprivation index, engaged in more daily exercise, accumulated lower total weekly MET minutes across all activities, had lower BMI, higher rates of alcohol consumption and current smoking, and higher prevalence of hypertension, diabetes, and COPD.

**Table 1:** Baseline characteristics according to activity patterns.

Characteristics	Activity patterns				
	Morning	Evening	Mixed	Midday-afternoon	P-value
Number of participants	7223	3352	13815	20082	
Age (years)	56.4(7.7)	52.9(7.7)	53.4(7.7)	56.4(7.4)	<0.001
Race[N, (%)]					0.945
White	6711(92.9)	3004(89.6)	12372(89.6)	18504(92.1)	
Non-white	493(6.8)	336(10.0)	1413(10.2)	1524(7.6)	
Qualifications [N, (%)]					0.839
College or University degree	2689(37.2)	1509(45.0)	6418(46.5)	8349(31.6)	
Below college or University degree	3789(52.4)	1647(49.2)	6431(46.6)	9871(49.2)	
None of above	676(9.4)	167(5.0)	827(6.0)	1666(8.3)	
The missing value	69(0.96)	29(0.9)	139(1.0)	196(1.0)	
Townsend deprivation index	-1.8(3.4)	-1.4(5.1)	-1.3(4.7)	-1.7(4.3)	0.532
BMI (kg/m²)[N, (%)]					<0.001
<25kg/m²	3123(43.2)	1529(45.6)	7205(52.2)	9400(46.8)	
25-29.9kg/m²	2670(37.0)	1157(34.5)	4561(33.0)	7191(35.8)	
≥30kg/²	1430(19.8)	666(19.9)	2049(14.8)	3491(17.4)	
Alcohol intake frequency					0.555
Daily or almost daily	1366(18.9)	565(16.9)	2501(18.1)	3927(19.6)	
Three or four times a week	1628(22.5)	816(24.3)	3500(25.3)	4842(24.1)	
Once or twice a week	1833(25.4)	853(25.4)	3674(26.6)	5054(25.2)	
One to three times a month	896(12.4)	495(14.8)	1769(12.8)	2515(12.5)	
Special occasions only	953(13.2)	431(12.9)	1553(11.2)	2425(12.1)	
Never	543(7.5)	191(5.7)	800(5.8)	1298(6.5)	
Smoking status[N, (%)]					0.025
Never	4358(60.3)	2092(62.4)	8676(62.8)	12310(61.3)	
previous	2445(33.9)	1045(31.2)	4328(31.3)	6628(33.0)	
Current	396(5.5)	209(6.2)	781(5.7)	1085(5.4)	
Summed MET minutes per week for all activity (minutes)	2575.0(2239.0)	2267.9(1990.8)	2486.0(2120.7)	2477.5(2134.5)	0.260
Total daily exercise time(minutes)	58.7(14.5)	56.6(14.0)	64.7(15.7)	59.1(14.2)	0.002
Current hypertension [N, (%)]	1599(19.8)	541(16.1)	1979(14.3)	3683(18.3)	0.228
Current diabetes [N, (%)]	63(0.8)	23(0.7)	78 (0.6)	131(0.7)	0.842
Current COPD [N, (%)]	34(0.4)	11(0.3)	57(0.4)	102(0.5)	0.123

Continuous variables are presented as mean (SD, standard deviation). Categorical variables are presented as numbers (%, percentage). BMI, body mass index. MET, Metabolic Equivalent Task. COPD, chronic obstructive pulmonary disease.

### 3.2 Association between activity patterns and hazard of malignant neoplasms of female genital organs

Multivariable Cox proportional hazards regression models were used to examine the relationship between activity patterns and the risk of malignant neoplasms of female genital organs, with results shown in **Table 2**. After adjusting for age, ethnicity, education, Townsend deprivation index, BMI, smoking status, moderate alcohol consumption, daily total exercise time, total weekly MET minutes for all activities, and history of diabetes, hypertension, and COPD, we found that women engaging in evening or mixed-time exercise had a significantly elevated risk of malignant neoplasms of female genital organs (HR evening = 1.530, 95% CI: 1.006–2.326; HR mixed = 1.406, 95% CI: 1.026–1.928, *P* for trend <0.05). These associations remained consistent from Model 1 to Model 4. Sensitivity analyses also confirmed the stability of the relationship between activity patterns and malignant neoplasms of female genital organs (**Table 3**). Furthermore, conclusions remained largely unchanged after adjusting for diabetes and COPD status. After excluding women diagnosed with malignant neoplasms of genital organs within one year and two years of follow-up, the associations between evening and mixed-time exercise and malignant neoplasms of female genital organs remained stable. The hazard trend for midday-afternoon exercise on malignant neoplasms of female genital organs remained but lost statistical significance, possibly due to the smaller sample size of women with these malignancies.

**Table 2:** Hazard ratios and 95% confidence intervals for activity patterns with the hazard of female reproductive diseases.

Activity patterns	Evening	Mixed	Midday-afternoon	Morning	P _for trend_
Cases/N	36/3352	129/13815	196/20082	58/7223	
M1	1.328(0.876,2.013)	1.151(0.844,1.568)	1.222(0.912,1.638)	Ref	0.179
M2	1.553(1.022,2.360)	1.319(0.964,1.803)	1.225(0.914,1.643)	Ref	0.039
M3	1.528(1.005,2.324)	1.410(1.028,1.932)	1.256(0.936,1.684)	Ref	0.033
M4	1.530(1.006,2.326)	1.406(1.026,1.928)	1.254(0.935,1.682)	Ref	0.034

Data are HRs and 95%CI;

(M1) Model 1 was not adjusted;

(M2) Model 2 was adjusted for age, race, education, Townsend deprivation index;

(M3) Model 3 was model 2 with additional adjustments for BMI, smoke status, alcohol intake frequency, total daily exercise time, summed MET minutes per week for all activity;

(M4) Model 4 was model 3 with additional adjustments for histories of diabetes, hypertension, COPD.

Malignant tumors of the female reproductive system include malignant tumors of the vulva, malignant tumors of the vagina, malignant tumors of the uterine cervix, malignant tumors of the uterine corpus, malignant tumors of the uterus(site unspecified), malignant tumors of the ovary, malignant tumors of the placenta, and other unspecified malignant tumors of the female reproductive system.

**Table 3:** Sensitivity analyses for association between activity patterns and hazard of female reproductive diseases.

Activity patterns	Midday-afternoon	Evening	Mixed	Morning	P _for trend_
Excluding participants diagnosed with diabetes	1.528(1.005,2.323)	1.391(1.015,1.907)	1.240(0.924,1.663)	Ref	0.040
Excluding participants diagnosed with COPD	1.479(0.969,2.257)	1.389(1.013,1.905)	1.240(0.925,1.664)	Ref	0.041
Excluding participants diagnosed with female reproductive diseases within one year of follow-up	1.529(1.005,2.324)	1.389(1.012,1.905)	1.248(0.930,1.673)	Ref	0.042
Excluding participants diagnosed with female reproductive diseases within two year of follow-up	1.492(0.944,2.358)	1.468(1.044,2.065)	1.245(0.905,1.714)	Ref	0.027

Results were adjusted for age, race, education, Townsend deprivation index, BMI, smoke status, alcohol intake frequency, total daily exercise time, summed MET minutes per week for all activity, histories of diabetes, hypertension, COPD at baseline.

Malignant tumors of the female reproductive system include malignant tumors of the vulva, malignant tumors of the vagina, malignant tumors of the uterine cervix, malignant tumors of the uterine corpus, malignant tumors of the uterus(site unspecified), malignant tumors of the ovary, malignant tumors of the placenta, and other unspecified malignant tumors of the female reproductive system.

### 3.3 Association between activity patterns and hazard of malignant neoplasms of female genital organs stratified by potential risk factors

We also conducted stratified analyses based on potential risk factors including age, ethnicity, education, Townsend deprivation index, smoking status, and alcohol consumption frequency, as shown in **Table 4**. No hazardous effect of evening or mixed-time exercise on malignant neoplasms of female genital organs was observed among participants aged <50 years, while no hazardous effect of evening exercise was observed among those aged ≥50 years (interaction P = 0.002). No significant interactions were found between other potential confounders and activity patterns regarding the hazard of malignant neoplasms of female genital organs.

**Table 4:** Stratified analyses for association between activity patterns and hazard of female reproductive diseases.

Activity patterns	Evening	Mixed	Midday-afternoon	Morning	P _trend_	P _for interaction_
Age at baseline						0.002
<50 years old	2.105(0.761,5.823)	0.978(0.384,2.493)	1.430(0.582,3.518)	Ref	0.152	
≥ 50 years old	1.379(0.859,2.213)	1.504(1.077,2.100)	1.227(0.899,1.673)	Ref	0.017	
BMI						0.030
<25 kg/m^2^	0.81(0.61,1.09)	0.97(0.71,1.31)	0.80(0.65,0.98)	Ref	0.045	
25-29.9 kg/m^2^	0.94(0.68,1.29)	1.13(0.80,1.59)	0.74(0.57,0.95)	Ref	0.040	
≥30 kg/m^2^	0.61(0.38,0.97)	0.96(0.62,1.51)	0.86(0.63,1.19)	Ref	0.513	
Race						0.880
White	1.533(0.985,2.385)	1.493(1.076,2.074)	1.259(0.926,1.711)	Ref	0.017	
Non-white	1.517(0.372,6.180)	0.810(0.240,2.735)	1.341(0.450,3.995)	Ref	0.561	
Townsend deprivation index						0.593
<median	1.224(0.706,2.122)	1.293(0.873,1.914)	1.228(0.861,1.752)	Ref	0.199	
≥median	2.186(1.120,4.266)	1.634(0.953,2.801)	1.306(0.775,2.201)	Ref	0.022	
Smoking status						0.875
Never	1.256(0.728,2.168)	1.239(0.833,1.842)	1.148(0.796,1.656)	Ref	0.290	
Ever	2.253(1.129,4.495)	1.878(1.090,3.234)	1.472(0.876,2.472)	Ref	0.021	
Current	0.959(0.084,10.889)	0.349(0.030,4.030)	1.196(0.245,5.829)	Ref	0.399	
Qualifications						0.363
College or University degree	1.505(0.805,2.815)	1.280(0.789,2.075)	1.133(0.719,1.787)	Ref	0.201	
Below College or University degree	1.605(0.857,3.004)	1.527(0.953,2.445)	1.548(1.006,2.383)	Ref	0.047	
None of above	1.606(0.418,6.168)	1.397(0.558,3.497)	0.612(0.249,1.505)	Ref	0.285	
Alcohol intake frequency						0.845
Daily or almost daily	1.983(0.626,6.281)	2.025(0.858,4.779)	2.146(0.964,4.779)	Ref	0.062	
Three or four times a week	0.661(0.218,2.007)	1.065(0.565,2.007)	1.140(0.639,2.033)	Ref	0.465	
Once or twice a week	1.001(0.389,2.575)	1.539(0.849,2.790)	0.798(0.440-1.446)	Ref	0.156	
One to three times a month	7.522(2.112,26.787)	2.413(0.683,8.523)	4.131(1.270-13.437)	Ref	0.002	
Special occasions only	2.166(0.772,6.079)	1.744(0.744,4.087)	1.008(0.443,2.295)	Ref	0.142	
Never	0.666(0.141,3.154)	0.671(0.244,1.847)	0.677(0.292,1.571)	Ref	0.364	

Results were adjusted for age, race, education, Townsend deprivation index, BMI, smoke status, alcohol intake frequency, total daily exercise time, summed MET minutes per week for all activity, histories of diabetes, hypertension, COPD at baseline.

Malignant tumors of the female reproductive system include malignant tumors of the vulva, malignant tumors of the vagina, malignant tumors of the uterine cervix, malignant tumors of the uterine corpus, malignant tumors of the uterus(site unspecified), malignant tumors of the ovary, malignant tumors of the placenta, and other unspecified malignant tumors of the female reproductive system.

### 3.4 Association between activity patterns and hazard of specific malignant neoplasms of female genital organs

Results from the multivariable Cox proportional hazards regression models for the associations between activity patterns and malignancies of the cervix, uterine corpus, ovaries, and other/unspecified female genital organs are presented in **Table 5**. After adjusting for potential confounders including age, ethnicity, education, Townsend deprivation index, BMI, smoking status, alcohol consumption frequency, daily total exercise time, total weekly MET minutes for all activities, and history of diabetes, hypertension, and COPD, the multivariable Cox regression models did not reveal statistically significant associations between activity patterns and malignancies of the cervix, uterine corpus, ovaries, or other/unspecified female genital organs.

**Table 5:** Hazard ratios and 95% confidence intervals for activity patterns with the hazard of specific female reproductive disease.

Diagnosis	Activity patterns	Evening	Mixed	Midday-afternoon	Morning	P _for trend_
Uterine corpus malignant tumor	Cases/N	19/3352	80/13815	102/20082	29/7223	
	M1	1.403(0.787,2.502)	1.428(0.934,2.184)	1.272(0.842,1.921)	Ref	0.398
	M2	1.642(0.918,2.937)	1.640(1.068,2.517)	1.267(0.839,1.915)	Ref	0.536
	M3	1.607(0.898,2.877)	1.800(1.169,2.772)	1.318(0.872,1.992)	Ref	0.404
	M4	1.606(0.897,2.875)	1.790(1.162,2.756)	1.313(0.869,1.985)	Ref	0.414
Cervical malignant tumor	Cases/N	2/3350	3/13815	6/20076	3/7220	
	M1	1.421(0.237,8.503)	0.516(0.104,2.556)	0.723(1.81,2.889)	Ref	0.510
	M2	1.467(0.240,8.963)	0.529(0.105,2.669)	0.740(0.185,2.959)	Ref	0.527
	M3	1.444(0.234,8.906)	0.552(0.108,2.813)	0.736(0.183,2.950)	Ref	0.532
	M4	1.429(0.231,8.821)	0.548(0.107,2.813)	0.732(0.182,2.933)	Ref	0.528
Ovarian malignant tumor	Cases/N	14/3352	37/13815	60/20082	21/7223	
	M1	1.426(0.725,2.804)	0.911(0.533,1.556)	1.034(0.629,1.699)	Ref	0.850
	M2	1.694 (0.857,3.348)	1.059(0.616,1.818)	1.047(0.637,1.721)	Ref	0.776
	M3	1.657(0.837,3.280)	1.120(0.649,1.931)	1.060(0.645,1.743)	Ref	0.829
	M4	1.659(0.838,3.284)	1.119(0.649,1.930)	1.059(0.644,1.742)	Ref	0.825
Other and unspecified malignant neoplasms of female genital organs	Cases/N	1/3352	7/13815	14/20082	3/7223	
	M1	0.713(0.074,6.850)	1.204(0.311,4.655)	1.690(0.486,5.880)	Ref	0.286
	M2	0.820(0.085,7.947)	1.349(0.345,5.278)	1.663(0.477,5.789)	Ref	0.338
	M3	0.829(0.085,8.074)	1.355(0.343,5.353)	1.665(0.478,5.806)	Ref	0.340
	M4	0.833(0.086,8.114)	1.359(0.344,5.368)	1.667(0.478,5.812)	Ref	0.340

Data are HRs and 95%CI;

Model 1 was not adjusted;

Model 2 was adjusted for age, race, education, Townsend deprivation index;

Model 3 was model 2 with additional adjustments for BMI, drink, smoke status, total daily exercise time, summed MET minutes per week for all activity;

Model 4 was model 3 with additional adjustments for histories of diabetes, hypertension, COPD.

Malignant tumors of the female reproductive system include malignant tumors of the vulva, malignant tumors of the vagina, malignant tumors of the uterine cervix, malignant tumors of the uterine corpus, malignant tumors of the uterus(site unspecified), malignant tumors of the ovary, malignant tumors of the placenta, and other unspecified malignant tumors of the female reproductive system.

## 4 Discussion

This study investigated the relationship between exercise timing patterns and malignant neoplasms of female genital organs. It found that, compared to the morning exercise group, women engaging in evening and mixed-time exercise had a significantly elevated risk of developing malignant neoplasms of the female reproductive system. Furthermore, in this prospective study comprising four separate cohorts, we found no association between activity patterns and malignancies of the cervix, uterine corpus, ovaries, or other/unspecified female genital organs. These associations were independent of the confounders included in the models, such as biological factors, lifestyle, socioeconomic status, and pre-existing diseases. The relationship between exercise patterns and malignant neoplasms of the female genital organs was also observed in sensitivity analyses, demonstrating the robustness of the results.

Previous human studies on the association between MVPA and gynecological cancers have often focused on single exercise interventions or short-term activity monitoring, yielding conflicting conclusions, particularly for cancers like ovarian cancer (14, 15). Most of these studies relied solely on self-reported leisure-time MVPA data to analyze its impact on the risk of ovarian and endometrial cancers (14–16). However, questionnaire-based methods struggle to distinguish unstructured MVPA in free-living settings (e.g., daily commuting, household chores) from structured exercise, potentially introducing recall bias and misclassification bias, leading to significant discrepancies in existing research findings. In contrast, our study utilized accelerometers to objectively capture comprehensive MVPA exposure data in free-living environments, encompassing key characteristics such as intensity, duration, and timing, which is crucial for accurately defining the long-term association between MVPA patterns and gynecological cancers. To date, only a few cohort studies have used accelerometers to assess the long-term impact of MVPA on cancer risk (17, 18), and prospective studies investigating specific MVPA patterns (e.g., exercise at specific times of day) and their association with an elevated risk of gynecological cancers have not been reported. Additionally, a 2019 case-control study suggested a potential association between lifetime leisure-time MVPA and ovarian cancer risk in premenopausal women (14), which aligns with our finding that specific MVPA patterns may have long-term hazardous effects on gynecological cancers.

Our study found that evening MVPA may be associated with a 1.530-fold higher risk of malignant neoplasms of female genital organs compared to morning exercise. The observation that evening and mixed-time exercise showed higher cancer risk associations than morning exercise aligns with theories of endogenous circadian rhythm regulation. Potential mechanisms may involve the following aspects. First, phase interference and amplitude dampening of the melatonin rhythm. Melatonin, a hormone secreted primarily at night, not only regulates sleep but has also been demonstrated to possess broad anti-cancer activity, including inducing apoptosis, inhibiting cell proliferation, reducing tumor growth and metastasis, mitigating side effects associated with chemotherapy and radiotherapy, reducing drug resistance in cancer treatment, and enhancing the efficacy of traditional anti-cancer therapies (19). A previous study indicated that daily exposure to bright light from 9:00 to 11:00 for two weeks led to a significant increase in the amplitude of core body temperature rhythm in elderly insomniacs. One possible explanation is that midday bright light alters brain serotonergic function, increasing melatonin secretion, a major metabolite of serotonin (5-HT) in the pineal gland (20). Repeated exposure to bright daytime light can effectively entrain circadian phase and increase the nocturnal plasma melatonin peak (21). Furthermore, evening exercise causes a significant phase delay in the nocturnal melatonin rise, potentially attenuating its crucial tumor-suppressive function, while morning exercise may improve nocturnal sleep quality by enhancing parasympathetic nervous activity (22). Previous studies have also shown other adverse effects of evening exercise, with later exercise times and higher intensities associated with delayed sleep onset, shortened sleep duration, reduced sleep quality, elevated nocturnal resting heart rate, and decreased nocturnal heart rate variability (23). The initiation and maintenance of healthy sleep function are characterized by parasympathetic nervous activity (e.g., decreased nocturnal resting heart rate and core body temperature, increased heart rate variability) (24, 25). Therefore, when parasympathetic reactivation is disrupted due to increased exercise intensity or later timing, sleep may be impaired in both timing and quality (23). Human core body temperature is higher during the day and decreases at night (26). Studies have shown that vigorous exercise close to bedtime simultaneously elevates pre-sleep core body temperature and heart rate, with the latter remaining elevated during sleep (27). Thus, nighttime exercise artificially elevates the core body temperature that should naturally begin to decline, directly opposing the natural rhythm. Circadian rhythms are crucial for processes like DNA repair and cell renewal. Such rhythm disruption has been linked to metabolic dysregulation, impaired immune function, and increased cancer risk (28). Insulin sensitivity (29) and glucose metabolism (30) also follow circadian rhythms, being higher during the day and lower at night. Previous research in mice suggests that low-intensity exercise before sleep is more effective than exercise after waking in reducing body weight, enhancing muscle strength and endurance, and improving glucose tolerance. This highlights the need for exercise to be synchronized with skeletal muscle circadian clock rhythms, otherwise exercise efficiency may be compromised (31). This indirectly suggests that engaging in moderate-to-vigorous intensity exercise during the rest period may conflict with intrinsic rhythms, thereby affecting metabolism. While exercise itself improves insulin sensitivity (32), performing it at an inappropriate time may disrupt metabolic rhythms in the long term, and hyperglycemia and insulin resistance are known cancer risk factors (33–35). Therefore, high-intensity exercise should be avoided in the evening.

Furthermore, we found that mixed-time MVPA may be associated with a 1.406-fold higher risk of malignant neoplasms of female genital organs. This may stem from its inability to provide a stable zeitgeber (time cue) input. Healthy circadian clock function relies on regular external cues. Mixed and irregular exercise times cannot effectively synchronize and reinforce circadian rhythms, leading the circadian system to remain in a state of long-term instability and desynchrony. This internal physiological chaos itself is an independent cancer risk factor. However, current research generally lacks direct evidence linking specific exercise timing to cancer risk, and we cannot draw definitive conclusions about the association between exercise timing and tumorigenesis. Therefore, the underlying biological mechanisms remain unclear and require further investigation.

We hypothesize that exercise timing may modulate cancer-related physiological pathways via circadian system interference. Research indicates that evening exercise may perturb the sleep-wake cycle, leading to delayed sleep onset, reduced sleep quality, and disrupted sleep architecture (23). Sleep disturbances and circadian disruption have been shown to promote hypothalamic-pituitary-adrenal (HPA) axis dysfunction (36), increase systemic inflammation and oxidative stress (37)—all known carcinogenic risk factors (38). Therefore, we hypothesize that evening exercise may potentially increase cancer risk by disrupting circadian homeostasis, impairing immune surveillance function, and creating a microenvironment conducive to tumorigenesis. Additionally, while mixed-time exercise might maintain some rhythmic stability, its effects could be attenuated by the irregularity of activity timing, failing to fully exert protective effects. However, this study lacked detailed data on participants’ sleep quality, melatonin levels, or inflammatory markers; therefore, the above hypotheses cannot be conclusively validated, and the underlying mechanisms require clarification in future research.

Subgroup analysis results indicated that the positive association between evening and mixed-time exercise patterns and elevated cancer risk was more pronounced among participants with a history of smoking. This suggests that smoking status may amplify the potential adverse effects of non-morning exercise. One possible explanation is that smoking itself represents a strong pro-inflammatory and pro-oxidative stress state (39–43), which could further exacerbate the metabolic dysregulation and immune dysfunction triggered by circadian disruption. When individuals are already in this “high-risk baseline” state, the additional disturbance to the circadian clock from evening exercise may have a synergistic effect, more markedly increasing cancer susceptibility.

Furthermore, substantial evidence confirms a causal association between alcohol consumption and the risk of various cancers (44–47). Alcohol can promote tumor development through multiple mechanisms, including acetaldehyde-mediated DNA damage, synergistic effects with tobacco, induction of chronic inflammation, disruption of hormonal balance (e.g., estrogen), and interference with epigenetics and metabolism (e.g., folate metabolism) (46). Therefore, besides carefully considering exercise timing, we recommend that the public, especially high-risk populations for cancer, should quit smoking and abstain from alcohol entirely to minimize the cancer risk posed by the superposition of various factors, including potentially inappropriate exercise timing. Additionally, it is noteworthy that direct evidence linking nighttime exercise to cancer risk is currently lacking, and research conclusions are inconsistent. Some studies suggest that afternoon or evening exercise may indirectly create an unfavorable metabolic environment for tumor growth by improving glycemic homeostasis (48). Moreover, evening exercise has been shown to significantly induce certain acute physiological responses, such as increased interleukin-6 (IL-6) and catecholamine levels, molecules that may possess dual potential for immunomodulation or pro-inflammation in specific contexts (49).

However, nighttime activity types (e.g., shift work) are significantly associated with an increased risk of cancers such as breast and prostate cancer (50–52). Although these studies focus on occupational circadian disruption, their findings support the broader hypothesis that disruption of central and peripheral circadian rhythms, including that potentially induced by nighttime exercise, may impair crucial tumor-suppressive pathways (53). Further research indicates that, compared to morning exercise, evening exercise may lead to circadian phase delay and reduced sleep quality and efficiency (23, 54). Sleep disturbances can promote appetite dysregulation and weight gain (55)and may induce a state of chronic low-grade inflammation (56), both of which are established cancer risk factors (57–61).

Despite these concerns, the relationship between evening exercise and cancer risk remains controversial. For example, a systematic review noted that healthy adults completing high-intensity exercise 2–4 hours before bedtime did not show significant negative effects on sleep quality (62). Although we controlled for overall exercise intensity (measured as MVPA) in our analysis, we cannot completely rule out other potential confounders, such as specific exercise duration, individual chronotype, or occupational light exposure, which constitutes a limitation of this study. Therefore, we currently find it difficult to make definitive claims about the true impact of nighttime exercise patterns on cancer risk.

Exploring mechanisms, exercise timing may influence core cancer-related physiological processes by modulating the circadian system. For instance, studies on female shift workers show that long-term circadian disruption is significantly associated with an increased risk of breast cancer (50, 51), with potential mechanisms possibly related to suppressed nocturnal melatonin secretion and consequent dysregulation of estrogen signaling pathways. Animal model studies further reveal that artificially induced jet lag accelerates tumor growth and alters the expression rhythms of key oncogenes and tumor suppressor genes (63).

At the metabolic level, processes like cell proliferation and DNA repair are highly dependent on circadian-regulated metabolic pathways, such as glycolysis, oxidative phosphorylation, and fatty acid oxidation (64–66). Global or tissue-specific (e.g., in liver or breast tissue) circadian disruption may lead to dysfunction in these metabolic networks, creating favorable conditions for tumorigenesis (67, 68).

Given that regular exercise is widely recognized as a cornerstone measure for primary cancer prevention, its temporal aspect deserves more attention. Lifestyle interventions, particularly integrating exercise into appropriate times of the day, may be especially important for high-risk populations (e.g., those with family history or obesity). Thus, we suggest that the public—particularly those concerned with cancer prevention—consider prioritizing morning exercise to potentially enhance health benefits through circadian rhythm stabilization. Furthermore, our results indicate no observed significant association between activity patterns and certain cancer types (e.g., endometrial and cervical cancers). This finding may relate to the specific etiology of these cancers. Taking cervical cancer as an example, its development is the result of a complex interplay of genetic factors, viral infection, hormonal, and metabolic factors (69–71). Specifically, metabolic abnormalities like obesity can increase HPV infection risk and disrupt the local microenvironment; HPV infection itself can activate specific metabolic pathways in cancer cells, creating a synergistic effect with metabolic disorders (72). More importantly, research reveals a strong positive additive interaction between endogenous hormones (e.g., high levels of estradiol E_1_) and HPV infection, with risk surging when both coexist, suggesting that hormonal imbalance and metabolic disturbances can synergize with HPV infection to drive malignant transformation (73). Under the dominance of these powerful core carcinogenic factors, the potential regulatory effects of exercise timing may be relatively easily masked. The etiology of ovarian cancer is even more complex, potentially requiring larger sample sizes to detect potential associations. Of course, the limited statistical power due to smaller sample sizes for some cancer types might also be a reason for the lack of observed significant associations. Currently, physical activity has been confirmed to exert protective effects against cancer through various mechanisms, including modulating sex hormone levels, improving insulin sensitivity, and enhancing immune function (74). However, existing evidence regarding the impact of exercise timing on more nuanced cancer biology processes, such as circadian gene expression and DNA repair efficiency, remains contradictory. On one hand, some studies suggest that evening exercise may indirectly exert anti-tumor effects by strengthening blood glucose control and lipid metabolism (75); on the other hand, its potential circadian-disrupting effects may also suppress nocturnal melatonin secretion, which is believed to have important tumor-suppressive functions (76).

Finally, although this study controlled for numerous potential confounders to the extent possible, some key variables were not included in the analysis due to data unavailability. Among these, chronic sleep disorders and long-term hormone replacement therapy history are two particularly noteworthy factors. Sleep deprivation or poor quality is known to influence cancer risk through various pathways, including disrupting circadian rhythms, promoting systemic inflammation, and impairing immune function. Simultaneously, exogenous hormone intake (e.g., hormone replacement therapy or oral contraceptives) has clear effects on the risk of hormone-related cancers (e.g., ovarian, endometrial cancer) and may interact with the physiological effects of exercise. Therefore, failure to comprehensively control for sleep quality and hormonal medication use may confound the true association when examining the relationship between exercise timing and cancer risk. Future research should systematically collect and assess these factors to more clearly elucidate the role of exercise timing in cancer prevention and provide robust evidence for formulating highly personalized public health strategies.

Our study provides important epidemiological evidence for ‘exercise chronotherapy.’ This is not just about ‘whether to exercise’ but also about ‘when to exercise.’ The optimal exercise time may be when it acts as a zeitgeber, achieving synergistic effects with the body’s internal biological clock rather than causing antagonistic interference. For populations with prevalent late-sleep/late-rise habits (“night owls”) or shift workers, whose circadian clocks are inherently fragile, evening exercise may further exacerbate their rhythm disruption, partially explaining the risk associations observed in this study.

Of course, interpretation of these results requires caution. First, individuals who exercise in the evening may inherently possess other underlying unhealthy lifestyle characteristics (e.g., higher stress levels, more irregular eating patterns). Although we endeavored to adjust for these, the possibility of residual confounding cannot be entirely excluded. Second, the assessment of physical activity relied on a single 7-day accelerometer measurement, which may not fully reflect participants’ long-term habitual activity patterns. Activity timing and volume can be influenced by short-term factors, such as seasonal variation, acute illness, work schedules, or temporary life events during the monitoring period, which could introduce non-differential measurement error into our exposure variable. Notably, the UK Biobank protocol required participants to wear the accelerometer for 7 consecutive days including both weekdays and weekends, which partially mitigates the impact of weekly activity variation. However, the absence of repeated measurements means we cannot account for potential long-term changes in activity behavior over the follow-up period. Future studies combining repeated accelerometer assessments or longer monitoring periods with circadian rhythm biomarkers (e.g., melatonin levels) would help to validate our findings.

The present findings have moderate generalizability. They are applicable to middle−aged and elderly women of European ancestry in developed countries, but may not be generalizable to men, younger individuals, non−white populations, or residents of low− and middle−income countries.

In summary, we speculate that evening and mixed-time exercise may attenuate the anti-cancer protective effects that exercise should confer, or even pose potential risks, potentially by interfering with melatonin secretion and disrupting core body temperature and metabolic rhythms. This finding highlights the potential importance of considering ‘exercise timing’ as a modifiable lifestyle factor in public health guidelines, alongside exercise frequency, intensity, and duration. Future research should strive to provide personalized optimal exercise time recommendations for individuals with different daily rhythms habits (e.g., morning types and evening types).

## 5 Conclusions

In summary, our findings suggest that evening or mixed-time moderate-to-vigorous physical activity (MVPA, e.g., brisk walking) is associated with a higher risk of malignant neoplasms of female genital organs compared with morning exercise. These results support prioritizing morning MVPA over evening or mixed-time MVPA for reducing the risk of female genital malignancies.

## Data Availability

The data analyzed in this study is subject to the following licenses/restrictions: The dataset is sourced from UK Biobank (publicly available database). Access is restricted by UK Biobank’s Data Access Agreement and Governance Policies. Researchers must apply for official authorization and complete ethical review to access the dataset, and it cannot be shared or distributed without prior written permission from UK Biobank. Requests to access these datasets should be directed to Tianshu Han, 102403@hrbmu.edu.cn.
